# 2324. COVID-19 Knowledge, Attitudes, and Practices Among People Living with HIV in Sub-Saharan Africa

**DOI:** 10.1093/ofid/ofad500.1946

**Published:** 2023-11-27

**Authors:** Brennan R Cebula, Allahna Esber, Nicole Dear, Valentine Sing’oei, Jonah Maswai, Michael Iroezindu, Emmanuel Bahemana, Hannah Kibuuka, Trevor A Crowell, Neha Shah, Julie A Ake

**Affiliations:** Walter Reed Army Institute of Research, Silver Spring, Maryland; Henry M. Jackson Foundation for the Advancement of Military Medicine, Bethesda, Maryland; Henry M. Jackson Foundation for the Advancement of Military Medicine, Bethesda, Maryland; Henry M. Jackson Medical Research International, Kisumu, Western, Kenya; Walter Reed Army Institute of Research, Silver Spring, MD, Silver Spring, Maryland; Henry Jackson Foundation for the Advancement of Military Medicine, Abuja, Federal Capital Territory, Nigeria; Henry Jackson Foundation for the Advancement of Military Medicine, Abuja, Federal Capital Territory, Nigeria; Makerere University Walter Reed Project, Kampala, Kampala, Uganda; Henry M. Jackson Foundation for the Advancement of Military Medicine, Bethesda, Maryland; Walter Reed Army Institute of Research, Silver Spring, Maryland; Walter Reed Army Institute of Research, Silver Spring, Maryland

## Abstract

**Background:**

Infection control practices are imperative to mitigate COVID-19 transmission, and adherence likely depends on accurate understanding of their effectiveness along with the risks associated with COVID-19 infection. This is particularly important in people living with HIV (PLWH) as they may be at increased risk for severe disease and worse outcomes. There is limited understanding of the knowledge, attitudes, and practices (KAP) regarding COVID-19 risks and infection control measures among PLWH in sub-Saharan Africa.

**Methods:**

A questionnaire with Likert scale responses for COVID-19 knowledge/attitudes and binary responses for practices was administered beginning Mar 2022 to participants enrolled in the African Cohort Study (AFRICOS), a prospective cohort of adults and adolescents living with or at risk for HIV in Kenya, Nigeria, Tanzania, and Uganda. Multivariable logistic regression with generalized estimating equations was used to estimate odds ratios (OR) and 95% confidence intervals (CI) for associations between demographics and COVID-19 knowledge/attitudes with self-reported facemask use and social distancing in the preceding month.

**Results:**

As of 1 Dec 2022, 962 PLWH enrolled in AFRICOS had COVID-19 KAP data. Facemask use was reported by 784 participants (81.5%) and social distancing by 600 (62.4%). Compared to participants in Uganda, participants in Kenya or Tanzania had lower odds of facemask use and social distancing (Table 1). Participants with lower self-assessed general health status and who were less concerned about the spread of COVID-19 had lower odds of facemask use or social distancing. Participants who more highly agreed that facemasks reduce the risk of COVID-19 had higher odds of facemask use. Compared to 176 participants not living with HIV, PLWH had higher odds of facemask use and social distancing.

**Figure:**
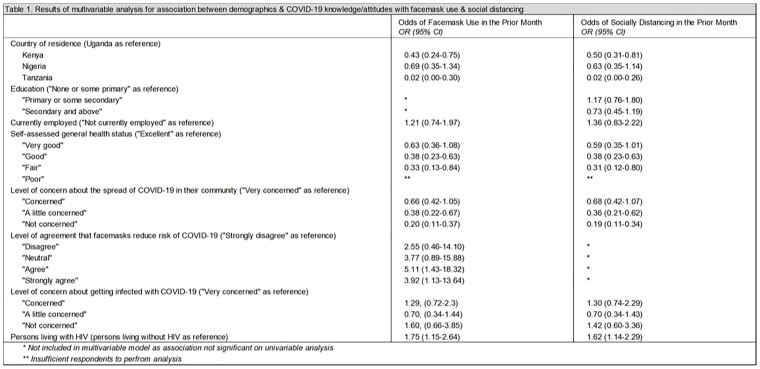

**Conclusion:**

These results provide important insight into COVID-19 KAP among PLWH in sub-Saharan Africa and suggest potential population and messaging targets to enhance future COVID-19 infection control efforts.

The views expressed are those of the author(s) and do not reflect the official policy of the Department of the Army, Department of Defense, or U.S. Government. The investigators adhered to policies for protection of human subjects prescribed in AR 70–25.

**Disclosures:**

**All Authors**: No reported disclosures

